# Identification of the Emerging C1-like Lineage of Enterovirus A71 in Two Uruguayan Children with Hand-Foot-and-Mouth Disease and Neurological Complications

**DOI:** 10.3390/v16111752

**Published:** 2024-11-08

**Authors:** Andrés Lizasoain, Natalia Martínez, Carla de Mora, Edivia Rodríguez, Nathalie Ledezma, Rodney Colina

**Affiliations:** 1 Molecular Virology Lab, Department of Biological Sciences, Centro Universitario Regional Litoral Norte, Universidad de la República, Salto 50000, Salto, Uruguay; 2 Centro de Asistencia Médica de Young, CAMY-IAMPP, Young 65100, Río Negro, Uruguay

**Keywords:** viral encephalitis, enterovirus A71, hand-foot-and-mouth disease, preschooler, neurological symptoms

## Abstract

Enterovirus A71 (EV-A71) is a major cause of hand-foot-and-mouth disease (HFMD), particularly in cases that involve complications affecting the nervous system or cardiopulmonary function. In South America, EV-A71 has primarily been identified through studies of acute flaccid paralysis (AFP) and other neurological disorders. In September 2022, two children from a small city in Uruguay were hospitalized with presumptive rhombencephalitis, exhibiting symptoms of HFMD. EV-A71 was identified through RT-PCR and next-generation sequencing of stool and skin lesion samples. A maximum-likelihood phylogenetic analysis of the P1 coding region classified the Uruguayan strains as part of an emerging lineage, primarily reported in Europe over the past decade, known as the C1-like lineage. The findings presented here represent the first detection of the EV-A71 C1-like lineage in cases of HFMD and encephalitis reported from South America, underscoring the urgent need to enhance surveillance for HFMD, aseptic meningitis, encephalitis, and AFP, in countries facing challenges in establishing effective surveillance programs related to enteroviruses and associated diseases.

## 1. Introduction

Enterovirus A71 (EV-A71) is a member of Species A within the Enterovirus genus, part of the *Picornaviridae* family [1]. This is a non-enveloped virus having a single-stranded positive-sense RNA genome of about 7 kb which contains a polyprotein coding region, that gives place to structural (VP1-VP4) and nonstructural (2A–2C and 3A–3D) proteins, and is flanked by a highly structured untranslated region (UTR) at 5′end, and by a shorter and highly conserved UTR at 3′end, followed by a final poly-A tail [2,3]. The virus is linked to a wide range of illnesses, with significant concern regarding those affecting the central nervous system, such as encephalitis, aseptic meningitis, paralysis, or myelitis [4]. However, EV-A71 is most commonly associated with hand-foot-and-mouth disease (HFMD) [5,6,7]. This is an acute, contagious disease characterized by mouth sores, anorexia, low-grade fever, and minor blisters or small ulcers on the hands, feet, and mouth. It primarily affects children under five years old, who generally recover spontaneously within about a week. However, HFMD can sometimes lead to complications involving the nervous or cardiopulmonary systems [7]. Additionally, infections caused by enterovirus A71 can present a serious threat to the patient’s life [8,9,10,11]. HFMD is linked to several enteroviruses, but EV-A71 has been classically a major cause of HFMD, with a preponderant role in cases with complications [6]. EV-A71 spreads through close personal contact with an infected individual, respiratory droplets from coughs or sneezes, the fecal–oral route, and contaminated surfaces [12]. Unlike in the Asia-Pacific region, where large annual epidemics of HFMD caused by EV-A71 are frequently reported, in other geographic regions, the virus circulation has been demonstrated just through the study of small outbreaks or sporadic cases [13]. Strains of EV-A71 are classified into several lineages and sub-lineages based on their genetic diversity at the VP1 coding region level [14,15]. There are eight genogroups (A to H), with genogroups B and C further divided into sub-genogroups (B0-B5 and C1-C5). Given the continuous evolution of EV-A71, it is crucial to monitor emerging variants from an epidemiological standpoint, as these variants could significantly impact child health and rapidly become dominant [16]. In South America, EV-A71 has been identified through studies of acute flaccid paralysis (AFP) cases and other nervous system disorders [17,18,19,20]. HFMD cases caused by this virus have been less frequently reported [21]. This article presents the first detection of an emerging variant of EV-A71 in Uruguay, identified through the analysis of samples from two cousins who were admitted to a pediatric unit with symptoms indicative of viral rhombencephalitis and lesions consistent with HFMD.

## 2. Materials and Methods

### 2.1. Description of the Cases

In September 2022, two children from a small city in Uruguay (Patient 1, preschooler, male, 20 months old. Patient 2, preschooler, female, 4 years old) were hospitalized after their parents sought emergency care due to general discomfort, several episodes of vomiting, axillary temperatures reaching values up to 39.2 °C and 39 °C (for Patient 1 and Patient 2, respectively), lower limb tremors, and ataxia. Patient 2 also presented dizziness and developed papules on the back of her hands, which progressed to vesicles. The children, who were cousins, had attended a party 60 h before Patient 1 presented to the emergency department. Patient 2 was admitted to the hospital 48 h later. The parents of both children stated that no other close relatives exhibited similar symptoms. Notably, Patient 1 had a history of treatment for low weight, while Patient 2 had experienced a runny nose and dry cough two weeks prior to the onset of the symptoms described. Twenty-four hours after admission, Patient 1 developed erythematous papules and vesicles on the hands and feet, as well as pharyngitis with vesicles, consistent with HFMD. Upon hospitalization, a physical examination and several tests were conducted. The results, summarized in Table 1, include findings from a Multiplex PCR Panel for CSF pathogens, based on CSF samples collected 12 and 24 h after the admission of Patient 1 and Patient 2, respectively. During their stay, both patients were started on treatment with acyclovir and ceftriaxone. After a neurologist confirmed the diagnosis of viral encephalitis for Patient 1, treatment was adjusted to continue only with acyclovir. For Patient 2, an otolaryngologic evaluation revealed acute sinusitis and otitis media, so antibiotic treatment was continued. Patient 1 showed improvement, with the fever subsiding by the third day of hospitalization and a reduction in ataxia and lower limb tremors. After six days, he was discharged with instructions for home follow-up. Patient 2 also had a favorable clinical course, with only slight lateralization of gait and no instability until the fourth day of hospitalization. A neurological evaluation confirmed the absence of fever and progressive improvement in ataxia, with no further vomiting. Patient 2 was discharged after five days, continuing treatment with oral amoxicillin-clavulanic acid and acyclovir.

### 2.2. Samples Collection, Preparation, and RNA Extraction

Given the presumptive diagnoses of viral encephalitis and HFMD, along with the epidemiological link between the two patients, pharyngeal swabs, skin lesion swabs, and stool samples were collected during the third day of hospitalization for Patient 1 and during the second day for Patient 2. These samples were sent to the Laboratorio de Virología Molecular at the Universidad de la República in Salto to investigate the presence of enteroviruses. A 10% fecal suspension was prepared in PBS 1X from 1 g of feces. Swab samples were collected using sterile dry polystyrene/polyester swabs (Deltalab Ltd., Barcelona, Spain) and eluted in PBS 1X. The eluates from the swabs and the fecal suspensions were then subjected to viral RNA extraction using the QIAamp^®^ Viral RNA Mini Kit (Qiagen^®^, Hilden, Germany), following the manufacturer’s instructions.

### 2.3. Enterovirus Detection

Detection of enteroviruses from swab eluates and fecal suspensions was carried out using a one-step RT-PCR with primers designed by Arita et al. [22], following the method described by Majumdar et al. [23]. These primers target a region of approximately 3900 bp of the enterovirus genome, annealing in the 5′ non-coding region (forward primer 5′NCR: 5′-AGTAGTAGCGATAGATTCGAGAT-3′) and in the CRE element sequence of the 2C protein-coding region (reverse primer CRE-R: 5′-GTAGCCGAGAGAGAGGGGATAGGA-3′). RT-PCR reactions were conducted using the SuperScript^TM^ III One-Step RT-PCR System with Platinum^TM^ Taq High Fidelity DNA Polymerase (Invitrogen^TM^, Waltham, MA, USA), adhering to the reagent concentrations and cycling conditions specified by Majumdar et al. [23]. The PCR products were resolved by electrophoresis on a 1% agarose gel, and bands of the expected size were visualized using a UV transilluminator. Positive and negative controls were included in each detection experiment. 

### 2.4. Next-Generation Sequencing

Bands of the expected size were excised from the gel and purified using the Zymoclean^TM^ Gel DNA Recovery Kit (Zymo Research Corp., Irvine, CA, USA) to prepare libraries with the Illumina DNA Prep Kit (Illumina Inc., San Diego, CA, USA), following the manufacturer’s recommendations. Sequencing was then performed using Illumina^®^ technology on an iSeq100^TM^ sequencing system (2 × 150 bp).

### 2.5. Sequencing Data Processing

FastQ files were inspected using the FASTQC tool (http://www.bioinformatics.babraham.ac.uk/projects/fastqc/, accessed on 20 September 2024). Raw reads were filtered for quality using a tool based on the FASTX-toolkit [24], retaining only those reads where more than 90% of the bases had a Phred quality score of 30 or higher. Additionally, Trimmomatic was employed to trim the first and last 10 bases from each read (151 bp) and to discard reads shorter than 100 bp. Sliding window trimming was also performed with a window size of 4 bp, requiring an average quality score of at least 15. De-replication was carried out using the derep-full length method of the VSEARCH software (Version 2.8.3) [25], removing reads with abundance values below 10. De-replicated forward and reverse reads were used to assemble de novo contigs of the amplified genomic region using SeqMan v7.0.0 from the Lasergene DNA Star package.

### 2.6. Phylogenetic Analyses

The full-length contigs obtained from Illumina sequencing were submitted to the Enterovirus Genotyping Tool [26] to identify the enterovirus type responsible for both cases. Phylogenetic reconstructions were performed based on (1) the full-length P1 coding region, (2) the full-length VP1 coding region, and (3) a partial segment of the VP1 coding region. Different sequences of strains related to the strains reported here were obtained by using the BLAST tool or were directly downloaded from the NCBI database together with reference sequences for different genetic lineages. The alignments of sequences were performed using MAFFT v7.0 [27], and phylogenetic reconstructions were conducted using the maximum-likelihood method under the General Time Reversible (GTR) substitution model with FastTree v2.1.11 [28], which employs an SH-like approach to estimate the statistical support for each node [29]. The resulting trees were visualized and edited using FigTree v1.4.4 (http://tree.bio.ed.ac.uk/software/figtree/, accessed on 22 September 2024).

### 2.7. Ethical Considerations

This study was approved by the Ethical Committee of CENUR Litoral Norte at the Universidad de la República (Exp. 311170-000168-23). Additionally, the hospital authorities where the children were treated granted permission to conduct the study, access the medical records of each patient, and publish the information and results. The parents signed a consent form to participate in this research and authorized physicians to access the children’s clinical histories to obtain the detailed information necessary for the clinical case descriptions.

## 3. Results

**Figure 1:**
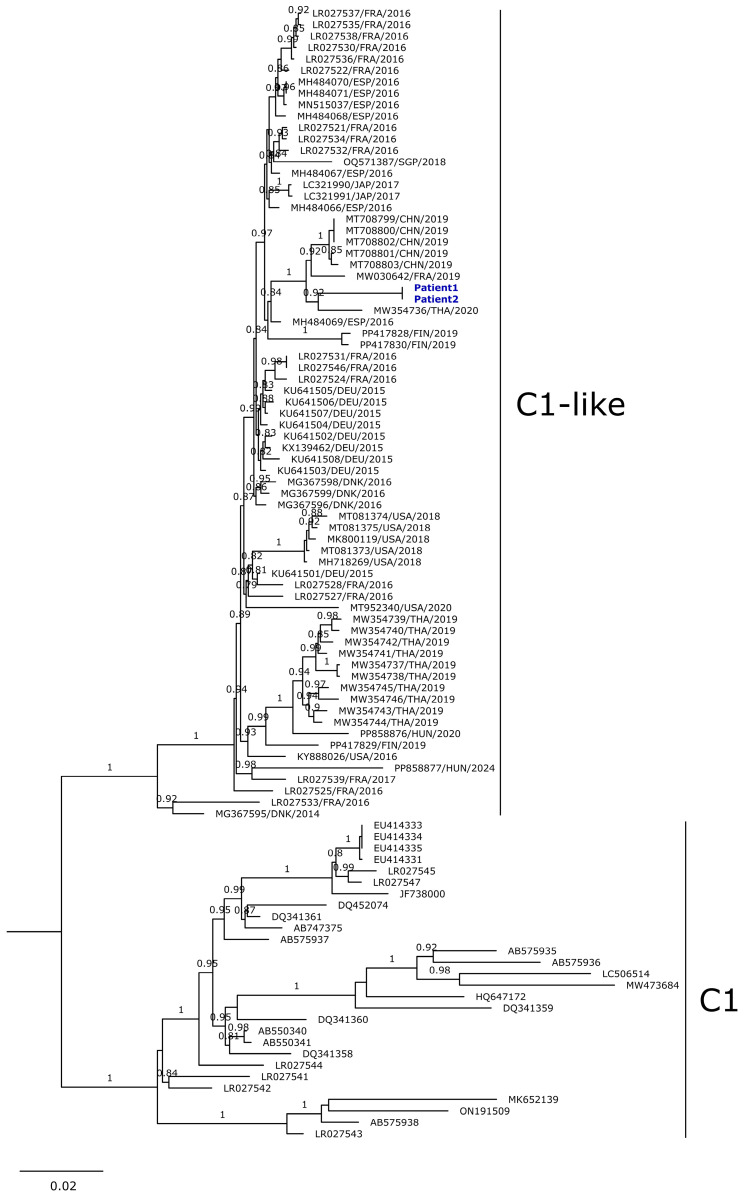
Maximum-likelihood phylogenetic tree of Enterovirus A71 C1-like strains. The tree was constructed using the General Time Reversible model and nucleotide sequences encoding the full-length P1 region from the two strains identified in this study, along with 69 sequences from C1-like strains earlier reported at the world level. Only SH-aLRT-like support values ≥ 0.75 are shown. The strains reported in this study from Patient 1 and Patient 2 are highlighted in blue. Twenty-eight sequences of the C1 genogroup were used as the outgroup. The bar at the bottom indicates genetic distance.

For Patient 1, both skin lesion swabs and stool samples tested positive for enterovirus detection via one-step RT-PCR. In the case of Patient 2, enterovirus detection was only possible in the stool sample. DNA bands of the expected size and of sufficient quality for sequencing were obtained from the skin lesion swab of Patient 1 and the stool sample of Patient 2. Next-generation sequencing experiments yielded contigs of 3890 and 3876 nucleotides in length for Patients 1 and 2, respectively. Using these contigs as queries in the Enterovirus Genotyping Tool revealed the presence of EV-A71. A phylogenetic analysis at the level of the complete VP1 coding region positioned the strains identified in this study within a sub-cluster closely related to the EV-A71-C1 sub-genogroup (SH-aLRT like support = 1). This sub-cluster—referred to as C1-like—includes strains primarily reported in Europe (Germany, Spain, Finland, Poland, Denmark, Hungary, and France) between 2014 and 2024, to a lesser extent in Asia (Japan, Taiwan, China, Singapore, and Thailand) between 2017 and 2020, and in the United Sates between 2016 and 2020. The Uruguayan strains from 2022 reported in this study clustered closely (SH-aLRT like support = 0.99) with strains reported between 2018 and 2020 in Germany, France, China, and Thailand (clade highlighted in red in Supplementary Figure S1). To analyze the relationship between the C1-like Uruguayan strains and two previously reported strains of the same lineage detected in wastewater samples collected in Argentina in 2017, we constructed an additional phylogenetic tree based on a 300-nucleotide partial fragment of the VP1 coding region (Supplementary Figure S2). This phylogeny indicates that the Argentine strains reported in 2017 cluster distinctly from the Uruguayan strains, belonging to two separate sub-clades. To gain a deeper understanding of the genetic divergence among the Uruguayan C1-like strains and previously reported C1-like strains worldwide, we conducted a phylogenetic reconstruction using nucleotide sequences of the entire P1 coding region, which is 2586 nucleotides long (Figure 1). The Uruguayan strains detected in Patient 1 and Patient 2 clustered closely with strains reported in France and China in 2019, as well as in Thailand in 2020, similar to what was observed when analyzing the full-length VP1 coding region. Additionally, a Spanish strain reported in 2016 (MH484069) was included in this sub-cluster (SH-aLRT like support = 0.84) during the analysis of the entire P1 region. When an identity matrix was calculated for the C1-like sequences shown in Figure 1, the Uruguayan strains were found to be between 93.1% and 97.1% identical at the nucleotide level compared to the other previously reported C1-like strains.

## 4. Discussion

This report represents the first documentation of EV-A71 detection in samples from patients with HFMD in Uruguay. It also marks the first instance in the country of HFMD cases linked to nervous system disorders. In South America, HFMD cases associated with EV-A71 are relatively rare [21,30], with coxsackievirus A6 (CVA6) typically playing a more prominent role in such clinical presentations [21,31,32,33,34,35]. Significant EV-A71 epidemics have predominantly occurred in the Asia-Pacific region. For instance, in China, HFMD has emerged as a major public health concern, with over 7 million cases reported during EV-A71 outbreaks [6]. Evidence of EV-A71 circulation in South America dates back to 1988–1990, when serum and/or fecal samples from patients with various nervous system disorders across Brazil revealed extensive viral spread [17,18,36]. In addition, Cisterna et al. [19] noted EV-A71 as a frequent cause of meningitis and AFP in Argentina from 1991 to 1998, identifying it as the third most detected enterovirus in 1993, following coxsackievirus B1 and echovirus 6. In recent years, Sousa et al. [20] reported that EV-A71 was responsible for approximately 8% of AFP cases in Brazil between 2005 and 2017. They observed that the B2 sub-genogroup was the predominant strain until 2014, when it was replaced by lineage C2. Less than a decade ago, several European countries began to report an increase in EV-A71 cases with severe clinical manifestations, coinciding with the emergence of a recombinant strain [37,38,39,40,41]. This novel variant formed a subcluster within the C1 sub-genogroup based on a VP1 sequence phylogenetic analysis and was designated as C1-like [37]. Since then, the global circulation of the C1-like lineage has been documented, both as a member of the diverse enterovirus serotypes in the environment and as a cause of HFMD and/or severe neurological disorders in children [42,43,44,45]. Given that this lineage has been circulating in South America since at least 2017, as indicated by our previous work with environmental samples from Argentina [43], and considering that case-based surveillance typically captures only 0.5% to 1% of enterovirus infections [42,46], it is unlikely that these two cases from 2022 are the first occurrences of encephalitis linked to EV-A71 C1-like in South America, despite this being the first reported instance. The Uruguayan strains detected in 2022 represent at least the third introduction of the C1-like virus into our geographic region, supporting the idea of a frequent influx from its epicenters in Europe and Asia. We compared the C1-like Uruguayan strains with previously reported C1-like viruses from various countries by analyzing both partial and full-length fragments of the VP1 coding region, as well as the entire P1 coding region. The strains from Patient 1 and Patient 2 consistently clustered with strains reported between 2019 and 2020 in France, China, and Thailand, exhibiting approximately 97% nucleotide similarity in the P1 region. This strongly suggests a genetic relationship with the recombinant C1-like variant that emerged in Europe in 2014–2015 and quickly spread worldwide. However, sequencing the complete genome of the C1-like Uruguayan strains could provide insights into additional recombination events that may have occurred during the silent circulation of the virus in South America. Unfortunately, we were unable to achieve this goal despite several attempts to amplify the missing regions of the genome. Although EV-A71 apparently could circulate silently for years in a given geographic region without causing nervous disorders that require medical attention [47], the five-year gap between the initial detection of the C1-like lineage in Argentina in 2017 and the identification of C1-like strains in two cases in Uruguay in 2022 likely reflects deficiencies and weaknesses in the surveillance systems of the South American region. For instance, an analysis of AFP surveillance indicators in the region suggests that some countries are still falling short of meeting the objectives set by the Global Polio Eradication Initiative defined by the WHO [48]. Uruguayan legislation mandates the investigation of suspected cases of viral meningitis, encephalitis, and poliomyelitis, as these are classified as health events that require immediate notification to authorities [49]. Additionally, there is a surveillance protocol for viral meningitis, encephalitis, and meningoencephalitis that involves studying CSF using molecular methods to detect various viruses, including enterovirus [50]. However, to the best of our knowledge, enterovirus typing is not routinely performed in hospitals or reference laboratories. Furthermore, there is a significant knowledge gap regarding enteroviruses associated with meningitis and encephalitis, as indicated by the limited published data from Uruguay, which mirrors the situation in other South American countries. As a result, South America has a low number of non-polio enterovirus genetic sequences available in GenBank, as previously noted by Brown et al. [51]. Although patients with EV-A71 infections might show normal results for the CNS in magnetic resonance imaging (MRI) [52], such imaging would have been a valuable addition to our diagnosis of viral encephalitis, which was primarily based on clinical examination. Additionally, when both CSF samples were assessed using a Multiplex PCR Panel for CSF Pathogens—including enteroviruses—the results were negative (Table 1). Considering that the detection of viral RNA in the CSF of patients with encephalitis can be inconsistent using PCR methods [53], performing viral isolation in a cell line could have offered an additional means to confirm the presence of EV-A71 in the CNS. Unfortunately, this was not performed and represents a limitation of our study, in addition to the absence of MRI data. This report on the EV-A71 C1-like lineage associated with illness in Uruguay underscores the need to improve surveillance for HFMD, aseptic meningitis, encephalitis, and AFP in a region where many countries have surveillance programs with weaknesses.

## Figures and Tables

**Table 1:** Overview of clinical and laboratory test findings during hospitalization.

		Patient 1	Patient 2
Examination at admission in the pediatric ward	PAT ^1^	stable	stable
	Heart Rate	118 beats per minute	106 beats per minute
	Respiratory Rate	26 breaths per minute	22 breaths per minute
	Temperature	37.6 °C	36.6 °C
	Blood glucose level	0.92 g/L	0.84 g/L
	Respiratory status	No respiratory distress; patient is well-perfused with a regular heart rhythm and no audible murmurs	No respiratory distress; patient is well-perfused with a regular heart rhythm and no audible murmurs
	Hemodynamics	Stable. Peripheral capillary refill time is 1 s. Pulses are full and strong	Stable. Peripheral capillary refill time is 2 s. Pulses are full and strong
	Pleuropulmonary examination	Clear bilateral air entry with no rales	Clear bilateral air entry with no rales
	Abdominal examination	Soft and compressible abdomen with no visceromegaly	Soft and compressible abdomen with no visceromegaly
	Neurological examination	Tone is appropriate. Symmetric muscular weakness in the lower extremities, with normal axial and peripheral tone. Motor coordination and cranial nerve functions are intact. Ataxic gait is noted, with no neck stiffness or purpuric petechial elements	Tone is appropriate, but there are tremors and muscular weakness in the lower limbs. Axial and peripheral tone are normal. Motor coordination is normal, as are the cranial nerves. The gait is ataxic, with no neck stiffness or purpuric petechial elements
	Others		There is mild dehydration, but the patient is well perfused; intravenous hydration was administered with a good response and adequate diuresis. The pharynx is congested, with hemorrhagic punctate and erythematous lesions. Scab-like lesions have developed on the back of the hands. Otoscopy reveals a bulging and erythematous right tympanic membrane
Laboratory assessments at admission in the pediatric ward	White Blood Cells Count	14,000/mm^3^ (62% neutrophils)	11,900/mm^3^ (67% neutrophils)
	Platelet Count	357,000/mm^3^	275,000/mm^3^
	C-Reactive Protein	6 mg/dL	ND ^2^
	Urine Drugs Screen	Negative	Negative
	Blood and Urine Cultures	No bacterial growth	ND
	Viral Respiratory Panel ^3^	Negative	Negative
	Lumbar Puncture ^4^	CSF ^5^ with normal pressure	CSF with normal pressure
	CSF Cytochemical Analysis	Pandy test positive; slight hypo-glycorrhachia; 278 WBC/mm^3^	Pandy test negative; 300 WBC/mm^3^
	CSF Culture	No bacterial growth	No bacterial growth
	Multiplex PCR Panel for CSF Pathogens ^6^	Negative	Negative
	Others	Liver Function Tests, Zymogram, Renal Function Tests, Electrolytes, and Coagulation Profile: all within normal limits	Liver Function Tests, Zymogram, Renal Function Tests, Electrolytes, and Coagulation Profile: all within normal limits
Imaging findings at admission in the pediatric ward	Computed Tomography of the Skull	Normal	Mucosal thickening observed at the level of the sphenoid sinuses and occupation of the ethmoid cells, predominantly on the left, consistent with acute sinusitis. No alterations in brain parenchyma or signs of intracranial hypertension were observed. The ventricular system is normal.

^1^ Pediatric Assessment Triangle. ^2^ Not done. ^3^ Detection of adenovirus, influenza A and B, parainfluenza, respiratory syncytial virus, and SARS-CoV-2. ^4^ The Lumbar Puncture was performed after to obtain the result of the Computed Tomography of the skull. ^5^ Cerebrospinal fluid. ^6^ Detection of human enterovirus, Parechovirus, Herpes simplex virus types 1 and 2, Varicella zoster virus, Epstein–Barr virus, Cytomegalovirus, *Listeria monocytogenes*, *Haemophilus influenzae*, *Streptococcus agalactiae*, and *Neisseria meningitidis.*

## Data Availability

The original data presented in the study are openly available in GenBank at PQ564660 and PQ564661.

## Supplementary Materials

The following supporting information can be downloaded at https://www.mdpi.com/article/10.3390/v16111752/s1, Figure S1: Maximum-likelihood phylogenetic tree of Enterovirus A71 C1-like strains based on the full-length VP1 coding region. Figure S2: Maximum-likelihood phylogenetic tree of Enterovirus A71 C1-like strains based on a partial segment of 300 nucleotides from the VP1 coding region.
